# Implementation of point-of-care EEG in a pediatric emergency department: a quality improvement study

**DOI:** 10.1007/s00431-025-06404-1

**Published:** 2025-09-28

**Authors:** Leopold Simma, Katharina Moser, Michelle Seiler, Georgia Ramantani, Bigna K. Bölsterli

**Affiliations:** 1 Emergency Department, University Children’s Hospital Zurich, University of Zurich, Lenggstrasse 30, CH-8008 Zurich, Switzerland; 2 Children’s Research Center, University Children’s Hospital Zurich, University of Zurich, Zurich, Switzerland; 3 Department of Neuropediatrics, University Children’s Hospital, University of Zurich, Zurich, Switzerland; 4 Department of Pediatrics and Adolescent Medicine, Division of Neonatology, Intensive Care and Neuropediatrics, Medical University of Vienna, Vienna, Austria; 5 Department of Pediatric Neurology, Children’s Hospital of Eastern Switzerland, St. Gallen, Switzerland; 6 Child Development Center, University Children’s Hospital Zurich, University of Zurich, Zurich, Switzerland

**Keywords:** Simplified EEG, Emergency department, Altered mental status, Nonconvulsive status epilepticus, Status epilepticus, Electroencephalogram

## Abstract

**Abstract:**

Central nervous system disorders are among the most common reasons for pediatric emergency department (PED) visits. Status epilepticus (SE) and nonconvulsive status epilepticus (NCSE) are particularly concerning, and the latter requires electroencephalography (EEG) for diagnosis. However, standard EEG is resource intensive and rarely available outside regular working hours. Point-of-care EEG (pocEEG) is a novel tool for rapid neuromonitoring in the PED. We aimed to implement pocEEG as a quality improvement initiative in a tertiary pediatric hospital. A simplified two-channel EEG setup was gradually implemented in the PED. A convenience sample of patients was recruited to assess feasibility. The clinical data of 62 pocEEG recordings were retrospectively analyzed. Concordance was assessed with standard EEGs within 48 h. Abnormal findings were observed in 45% (28/62) of pocEEGs, more frequently in patients with known pre-existing conditions (18/28 vs. 10/28, *p* = .024). Seizure activity was recorded in 16% of cases (10/62), mostly in patients with pre-existing conditions (8/10). Concordance between pocEEG and standard EEG was assessed in 37/62 pocEEGs, of which 68% were concordant and 8% normalized before standard EEG. pocEEG influenced 60% of clinical decisions by aiding altered mental status (AMS) assessment, antiseizure medication guidance in active SE, and NCSE identification. *Conclusion*: pocEEG is a feasible and effective tool for rapid neuromonitoring in the PED. It aids seizure detection, AMS evaluation, and treatment decisions. Further research is warranted to assess its impact on time to diagnosis, seizure duration, outcomes, cost-effectiveness, and standardized workflows for timely standard EEG follow-up.

**Table:** 

What is Known: • Altered mental status (AMS) and seizures are frequent and high-acuity presentations in pediatric emergency critical care settings. • Nonconvulsive status epilepticus can only be diagnosed by EEG, yet immediate access to standard EEG is often unavailable.
What is New: • This quality improvement study shows that simplified, point-of-care EEG (pocEEG) can be successfully implemented and is a valuable tool for rapid neuromonitoring. • The study demonstrates feasibility of pocEEG with enhanced seizure detection and AMS assessment with the potential to bridge critical diagnostic gaps where 24/7 standard EEG is unavailable.

**Graphical Abstract:**

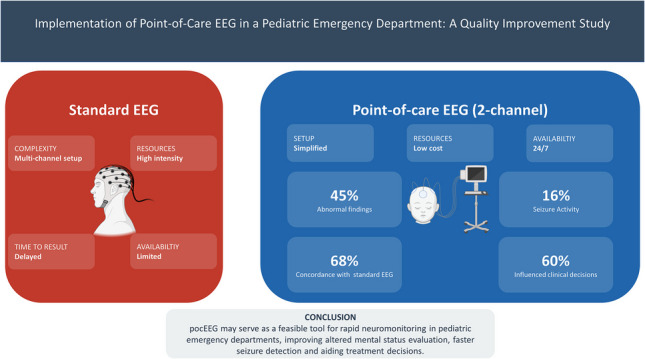

**Supplementary Information:**

The online version contains supplementary material available at 10.1007/s00431-025-06404-1.

## Introduction

Nontraumatic, acute central nervous system (CNS) disorders are among the most common pediatric emergencies [1], particularly in critically ill children [2, 3]. Convulsive status epilepticus (SE) is the most frequent neurological emergency [4]. In the emergency setting, distinguishing ongoing seizure activity from postictal states or non-epileptic causes of altered mental status is essential to guide appropriate escalation or de-escalation of treatment. Nonconvulsive SE (NCSE) is often under-recognized in encephalopathic patients despite its significant impact on outcomes and length of hospital stay [5, 6]. Early recognition of both SE and NCSE is essential to guide timely treatment and improve patient outcomes.

Electroencephalography (EEG) is the gold standard for detecting changes in cortical electrical activity in neurological emergencies, including altered mental status (AMS) [7]. EEG provides essential diagnostic information and supports clinical decision-making [8, 9]. However, standard EEG is rarely available in the PED outside regular working hours; is difficult to obtain during evenings, nights, and weekends; and carries significant costs [10]. Where standard EEG is unavailable, simplified bedside EEG in the PED has been shown to supplement clinical, laboratory, and imaging studies and assists in patient management [11, 12]. Point-of-care EEG (pocEEG) offers a rapid, accessible alternative for treatment monitoring of ongoing SE and timely NCSE detection and thus facilitates earlier intervention [13, 14].

A shortage of trained EEG personnel outside working hours is a common challenge in many centers and can limit timely EEG availability in critical cases. To address this gap at our tertiary pediatric institution, we introduced pocEEG [13] within a quality improvement (QI) project [14]. The primary objective was to improve patient care, and the secondary objective was to establish a database of pocEEG recordings for training PED personnel in basic EEG interpretation. We took an incremental approach by first ensuring technical feasibility [15] and then evaluating whether pocEEG could improve the management of children with AMS, including SE and NCSE, by enabling earlier recognition and intervention. While previous studies have described pocEEG use in adults and technical feasibility in selected pediatric cases, evidence is lacking on how pocEEG integrates into real-world pediatric emergency workflows and whether it meaningfully contributes to clinical decision-making. This study addresses that gap by evaluating whether simplified point-of-care EEG can be feasibly implemented in the PED and whether it supports clinical decision-making in children presenting with AMS, including suspected seizures.

## Methods

### Setting

This project was conducted in the PED of an academic, tertiary pediatric hospital with an annual census of approximately 50,000 patients. The neurophysiology team provides an EEG service on weekdays from 08:00 to 17:00, but EEG technicians are not on call or regularly available outside these hours. The PED team includes 21 attending pediatric emergency medicine (PEM) physicians, 6 fellows, 54 nurses, and 18 pediatric and pediatric surgery residents. The neuropediatrics team includes 10 attending physicians who cover the on-call roster for neurological emergencies.

### Intervention

We adapted a previously described setup [13] and enhanced it with EEG data and video recording [15]. The standard mobile patient monitor (Carescape B450, GE Healthcare, Finland) was fitted with an EEG module.

We used a simplified two-channel EEG to monitor brain activity by placing electrodes at F7/F8 (anterotemporal region) and approximate T5/6 (retroauricular/posterotemporal region) according to the 10–20 system with a median reference electrode on the forehead. Further technical details on pocEEG implementation can be found elsewhere [15]. PEM physicians received a 1-h face-to-face training session on pocEEG use and seizure pattern recognition. Implementation ran from January 25, 2021, to August 24, 2022. We recruited a convenience sample among patients who presented to the PED when trained senior medical staff were available.

### Study of the intervention

We collected all pocEEG recordings to assess signal quality and clinical utility in various clinical scenarios. PED staff interpreted the recordings ad hoc, and a neurophysiology fellow (KM) blinded to clinical data reviewed the recordings retrospectively. The American Clinical Neurophysiology Society’s Standardized Critical Care EEG Terminology [16] was used to guide interpretation.

### Measures

To assess quality improvement, we retrospectively analyzed pocEEG recordings and compared them with final discharge diagnoses, patient notes, and standard EEG reports available in electronic medical records. We assessed concordance between pocEEG and standard EEG. The standard EEG was used here as a surrogate reference when it was performed within 48 h of the pocEEG. However, we acknowledge that full concordance cannot be expected due to the sequential nature of the examinations.

### Analysis

We stored pocEEG recordings on a hospital notebook with regular backups to the hospital server. We collected data on patient demographics, known pre-existing conditions, PED arrival time, presenting symptoms, indication for pocEEG, chronic antiseizure medication (ASM) use, acute ASM treatment, and final discharge diagnoses. We coded patients using a modified International League Against Epilepsy (ILAE) classification and incorporated three non-epilepsy categories: acute symptomatic seizures, psychogenic nonepileptic seizures (PNES), and other non-epilepsy diagnoses. Standard EEG reports were retrieved from electronic medical records for comparison. We assessed concordance between pocEEG and standard EEG using dichotomous (normal, abnormal) and categorical (symmetry, epileptic discharges, seizure activity) variables.

Data were analyzed using with RStudio (R version 4.2.1, R Foundation for Statistical Computing, Vienna, Austria). We applied the *χ*^2^ test or Fisher’s exact test when *n* ≤ 5 and considered *p* < 0.05 statistically significant.

### Ethical considerations

We obtained verbal consent from patients and their parents, depending on age and mental status. The Cantonal Ethics Committee Zurich (KEK-ZH), Switzerland, conducted an expedited review of this quality improvement project and determined that no formal approval was required (Req-2022–00098), as this type of project does not fall within the scope of the Swiss Human Research Act. The project was carried out in accordance with local requirements for quality improvement projects, with appropriate attention to data protection and confidentiality. This manuscript follows the SQUIRE 2.0 reporting guidelines [17, 18].

## Results

**Fig. 1:**
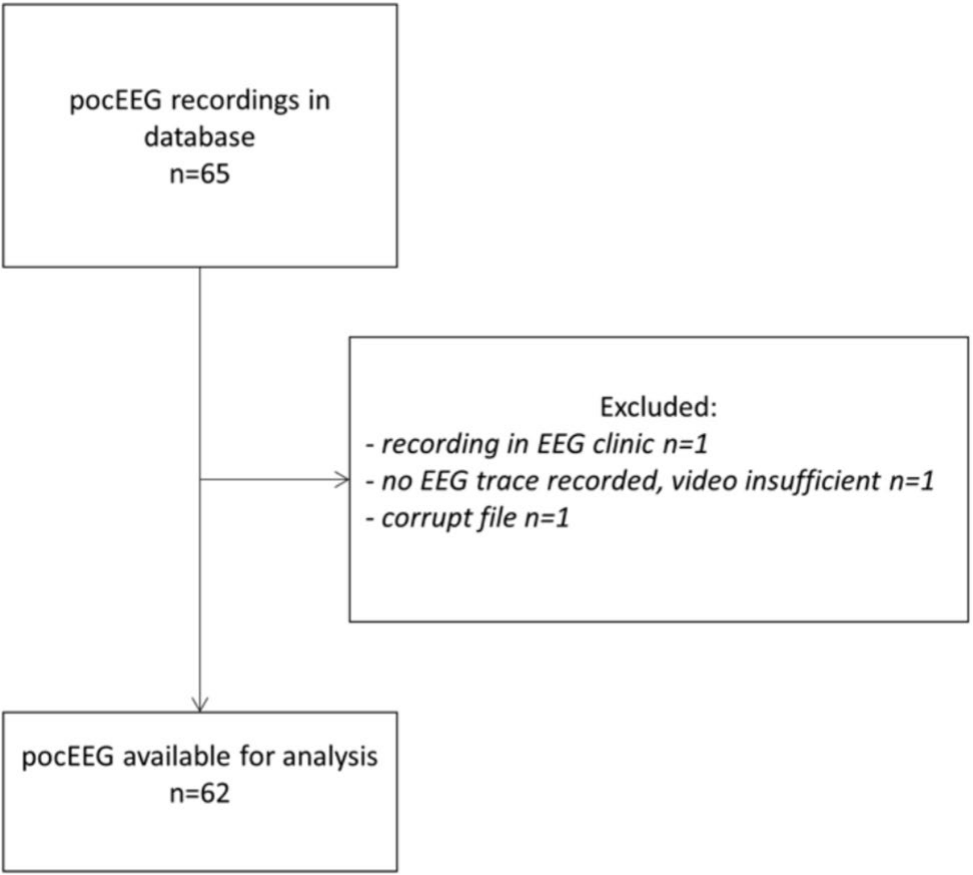
Flowchart pocEEG analysis

**Fig. 2:**
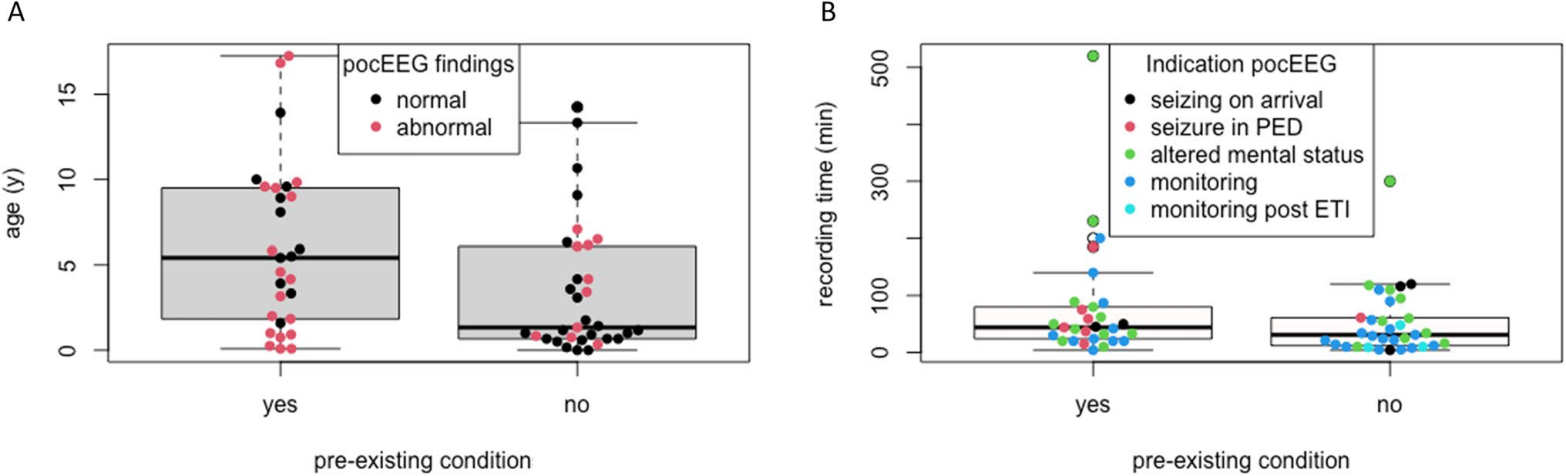
**A** Distribution of pocEEG findings by age and pre-existing condition. **B** Indication for neuromonitoring by pre-existing condition and recording length of pocEEGs. ETI endotracheal intubation, PED pediatric emergency department

We analyzed 62 pocEEG recordings from 62 patients (Fig. 1). Table 1 summarizes patient characteristics, and a detailed case list appears in Supplemental Table 1. In this cohort, 47% had pre-existing conditions and 26% were on chronic ASM. Self-limiting seizures were the most common reason for presentation and pocEEG application, occurring in 42% of cases. Another 27% presented with active SE, suspected NCSE, or a history of SE that had resolved before arrival at the PED. Half of the patients (50%) presented outside business hours, 55% required medical transport, and 40% had received acute ASM before arrival. Table 1 and Fig. 2B outline the indications for neuromonitoring. Overall, 79% (49/62) of patients were admitted to the hospital, and 19% (12/62) required pediatric intensive care unit (PICU) admission.

**Table 1:** Patient characteristics

	**Total**	**Pre-existing condition**		***p* value**
		**Yes**	**No**	
*n*	62 (100)	29 (46.8)	33 (53.2)	
Female (%)	21 (33.9)	10 (29.4)	11 (39.3)	0.716
Age at PED presentation in months (median [IQR])	40.5 (11.0 −83.3)	65 (22–114)	16 (8–73)	0.025^a^
Presenting symptoms (%)				0.075
Self-limiting seizure	9 (14.5)	3 (10.3)	6 (18.2)	
SE/NCSE	17 (27.4)	8 (27.6)	9 (27.3)	
Recurrent seizures	26 (41.9)	13 (44.8)	13 (39.4)	
Altered mental status	1 (1.6)	1 (3.4)	0 (0.0)	
Other symptom	9 (14.5)	4 (13.8)	5 (15.2)	
Arrival at PED out-of-hours (%)	31 (50.0)	17 (58.6)	14 (42.4)	0.309
Arrival mode (%)				0.245
Ambulance	33 (53.2)	13 (44.8)	20 (60.6)	
Walk-in	28 (45.2)	16 (55.2)	12 (36.4)	
Helicopter	1 (1.6)	0 (0.0)	1 (3.0)	
Prehospital seizures (%)				0.496
Yes	50 (80.6)	23 (79.3)	27 (81.8)	
No	6 (9.7)	2 (6.9)	4 12.1)	
Possible seizure	6 (9.7)	4 (13.8)	2 (6.1)	
Indication for pocEEG (%)				0.092
Seizing on arrival	5 (8.1)	2 (6.9)	3 (9.1)	
Seizure in PED	7 (11.3)	6 (20.7)	1 (3.0)	
Altered mental status	21 (33.9)	11 (37.9)	10 (30.3)	
Monitoring	26 (41.9)	10 (34.5)	16 (48.5)	
Monitoring post ETI	3 (4.8)	0 (0.0)	3 (9.1)	
Chronic ASM (%)				< 0.001
None	46 (74.2)	13 (44.8)	33 (100.0)	
One ASM	5 (8.1)	5 (17.2)	0 (0.0)	
Multiple ASM	11 (17.7)	11 (37.9)	0 (0.0)	
Prehospital ASM (%)				0.094
None	37 (59.7)	14 (48.3)	23 (69.7)	
ASM po/pr	9 (14.5)	7 (24.1)	2 (6.1)	
ASM in/iv	16 (25.8)	8 (27.6)	8 (24.2)	
1 st ASM @PED (%)				0.02
none	35 (56.5)	11 (37.9)	24 (72.7)	
ASM po/pr	13 (21.0)	8 (27.6)	5 (15.2)	
ASM iv	14 (22.6)	10 (34.5)	4 (12.1)	
2nd ASM @PED (%)				0.228
None	49 (79.0)	21 (72.4)	28 (84.8)	
ASM po/pr	5 (8.1)	2 (6.9)	3 (9.1)	
ASM iv	8 (12.9)	6 (20.7)	2 (6.1)	
3rd ASM @PED (%)				
None	58 (93.5)	27 (93.1)	31 (93.9)	
ASM po/pr	0 (0.0)	0 (0.0)	0 (0.0)	
ASM iv	4 (6.5)	2 (6.9)	2 (6.1)	
Patient disposition (%)				0.428
Discharge home	13 (21.0)	4 (13.8)	9 (27.3)	
Ward	37 (59.7)	19 (65.5)	18 (54.5)	
PICU	12 (19.4)	6 (20.7)	6 (18.2)	

*ASM* antiseizure medication, *ETI* endotracheal intubation, *in* intranasal, *iv* intravenous, *PED* pediatric emergency department, *PICU* pediatric intensive care unit, *po/pr* oral/rectal, *SE/NCSE* status epilepticus/nonconvulsive status epilepticus

^a^Wilcoxon rank-sum test

### Process measures

Senior PED physicians or nurses set up pocEEG in under 5 min, which allowed real-time visualization of EEG signals on the patient monitor. Feedback from senior PED physicians highlighted the need for additional training in basic pocEEG interpretation and illustrative pocEEG recordings. Initially, space constraints at the bedside led us to mount the recording laptop on the mobile patient monitor. Staff feedback prompted the addition of a video-recording feature later in the project.

### pocEEG and standard EEG findings

A total of 3968 min of pocEEG recordings were collected, with a median duration of 39 min (range: 4–520, IQR 20–79). Figures 2 A and B illustrate pocEEG indications and recording durations. Abnormal pocEEG findings were observed in 45% of cases.

A significantly higher prevalence was observed in patients with pre-existing conditions (64.3% vs. 35.7%,* p* = 0.024). Seizure activity was recorded in 16% (10/62) of cases, almost exclusively in patients with pre-existing conditions (8/10).

**Fig. 3:**
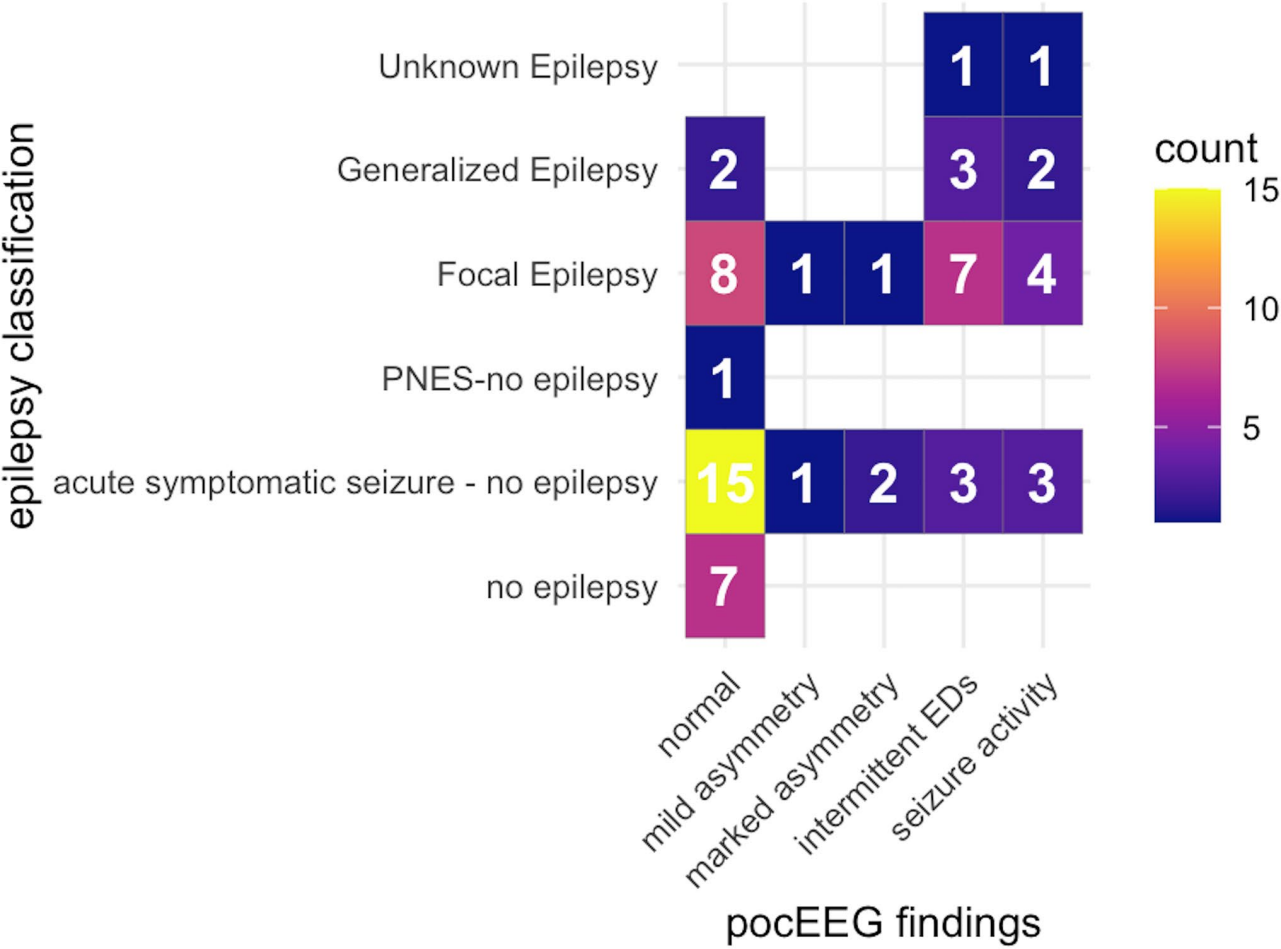
Heatmap with distribution of pocEEG findings among the modified ILAE epilepsy classes. PNES psychogenic nonepileptic seizures, EDs epileptic discharges

Table 2 provides an overview, and Fig. 3 presents findings by seizure and epilepsy type. Standard EEG was performed within 48 h in 37 (60%) cases and revealed abnormalities in 42% of patients. Recording quality on pocEEGs was generally good; 25 (40%) recordings had artifacts, but of these, only 6 (9.5%) contained sufficient artifacts to impede interpretation.

**Table 2:** Overview of pocEEG and standard EEG findings

		Pre-existing condition			
	Total	Yes	No		*p* value
*n*	62 (100)	29 (46.8)	33 (53.2)		
Epilepsy diagnoses (%)					< 0.001
No epilepsy	7 (11.3)	1 (3.4)	6 (18.2)		
Acute symptomatic seizure—no epilepsy	24 (38.7)	4 (13.8)	20 (60.6)		
PNES— no epilepsy	1 (1.6)	1 (3.4)	0 (0.0)		
Focal epilepsy	21 (33.9)	14 (48.3)	7 (21.2)		
Generalized epilepsy	7 (11.3)	7 (24.1)	0 (0.0)		
Unknown epilepsy	2 (3.2)	2 (6.9)	0 (0.0)		
pocEEG abnormal (%)	28 (45.2)	18 (62.1)	10 (30.3)		0.024
pocEEG findings (%)					0.006
Normal	33 (53.2)	11 (37.9)	22 (66.7)		
Mild asymmetry	2 (3.2)	0 (0.0)	2 (6.1)		
Marked asymmetry	3 (4.8)	0 (0.0)	3 (9.1)		
Interictal EDs	14 (22.6)	10 (34.5)	4 (12.1)		
Seizure activity	10 (16.1)	8 (27.6)	2 (6.1)		
Standard EEG < 48 h (%)					0.342
Stat (same day/in PED)	17 (45.9)	7 (46.7)	10 (45.5)		
24 h	11 (29.7)	6 (40.0)	5 (22.7)		
48 h	9 (24.3)	2 (13.3)	7 (31.8)		
Standard EEGs (%)					0.101
No sEEG < 48 h	25 (40.3)	14 (48.3)	11 (33.3)		
Normal	11 (17.7)	2 (6.9)	9 (27.3)		
Abnormal	26 (41.9)	13 (44.8)	13 (39.4)		
Standard EEG findings (%)					0.143
Normal	11 (29.7)	2 (13.3)	9 (40.9)		
Abnormal	14 (37.8)	6 (40.0)	8 (36.4)		
Interictal EDs	12 (32.4)	7 (46.7)	5 (22.7)		
Concordance sEEG—pocEEG (%)					0.79
No discrepancy	25 (67.6)	10 (66.7)	15 (68.2)		
Abnormality F-T leads	3 (8.1)	2 (13.3)	1 (4.5)		
Abnormality non F-T leads	6 (16.2)	2 (13.3)	4 (18.2)		
Normalized	3 (8.1)	1 (6.7)	2 (9.1)		

*EDs* epileptic discharges, *F-T* frontotemporal leads (F7/F8-T5/T6), *PED* pediatric emergency department, *PNES* psychogenic nonepileptic seizures, *sEEG* standard EEG

### Concordance between pocEEG and standard EEG

Post hoc analysis showed concordance between pocEEG and standard EEG in 25 of 37 cases (68%), with three cases (3/37 (8%)) normalizing before standard EEG, for example due to resolution of seizure activity or asymmetry. Six standard EEG recordings identified abnormalities outside the pocEEG-sampled region and three detected abnormalities within the pocEEG-sampled region. These cases included (1) three patients with acute symptomatic seizures due to gastroenteritis classified as normal on pocEEG but showing bilateral slowing on standard EEG, (2) two patients with pre-existing conditions who showed right fronto-temporo-parietal spikes on standard EEG within 24 h, and (3) one patient with right slowing on standard EEG within 48 h.

### Clinical impact of pocEEG

**Fig. 4:**
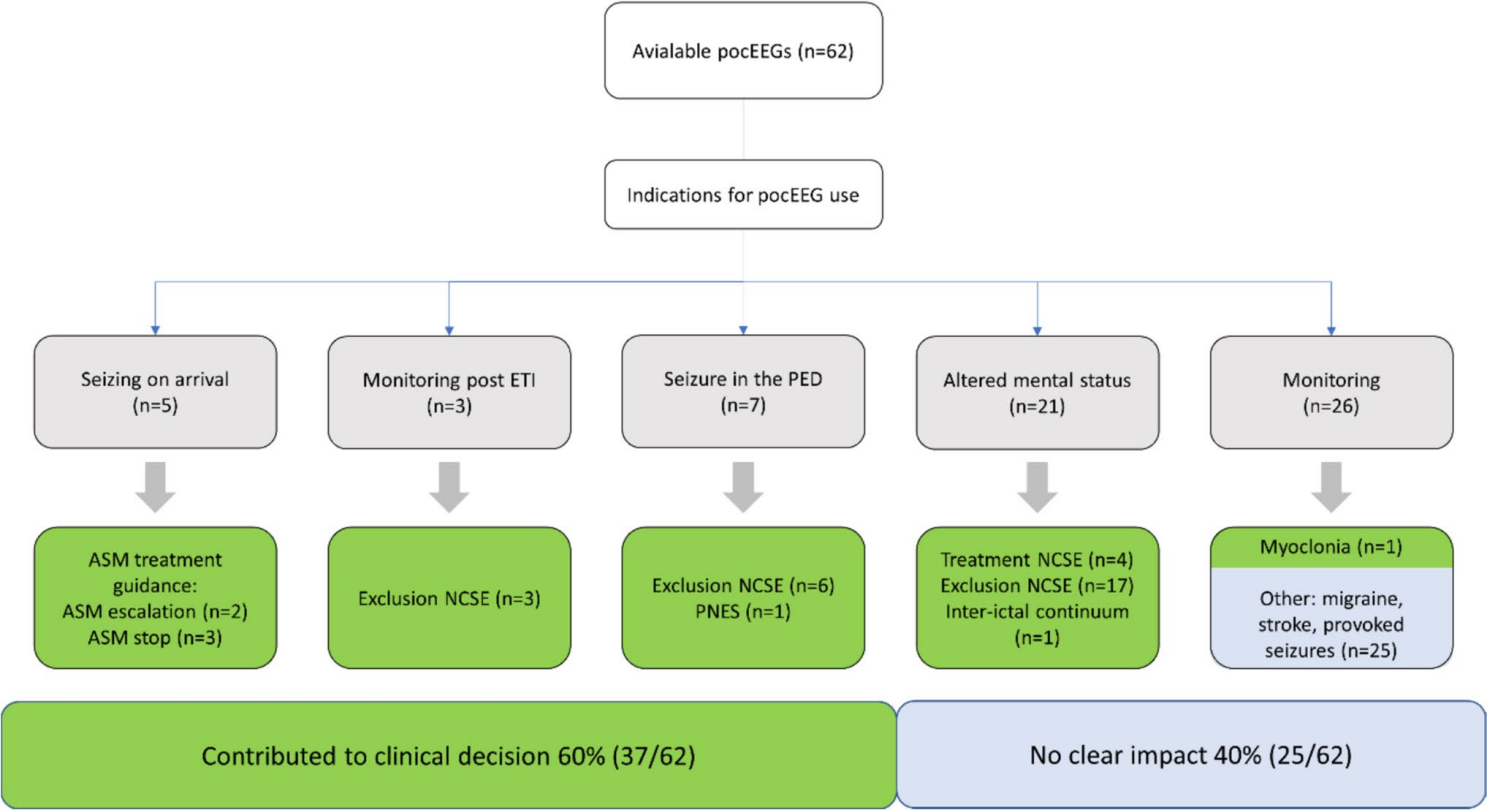
Clinical impact of point-of-care EEG (pocEEG) in 62 pediatric emergency department (PED) patients. In 37 cases (60%), pocEEG contributed to clinical decision-making by supporting the assessment of altered mental status, seizure detection (including exclusion of seizures in postictal states), or guiding treatment. This included escalation or discontinuation of antiseizure medication (ASM) in convulsive SE, diagnosis and management of nonconvulsive status epilepticus (NCSE), and identification of non-epileptic events such as myoclonia or psychogenic nonepileptic seizures (PNES)

The use of pocEEG influenced clinical decision-making in 37 (60%) patients, including assessment of AMS, seizure detection, and treatment guidance (Fig. 4; Table 1). Key contributions included assessment of AMS in 34%, onset of seizure activity in the PED in 11%, ongoing seizure activity on arrival in 8%, and exclusion of ongoing seizure activity after intubation for SE in 5% (Table 1). Final diagnoses are listed in Table 3 and Supplemental Table 1. Patients with seizure activity on pocEEG had a significantly higher likelihood of PICU admission (50% vs. 13.5%,* p* = 0.018). Two patients with active SE received ASM treatment under pocEEG monitoring. Four patients with NCSE were identified in the PED with pocEEG and successfully treated under neuromonitoring. One patient with AMS after a seizure at home had retrospective ictal-interictal continuum in the pocEEG and slowing in a first standard EEG, and a second standard EEG within 24 h of admission showed NSCE. In one patient with PNES, pocEEG findings helped de-escalate treatment and reassure the caregiver about the nonepileptic nature of the event despite the patient’s history of self-limiting focal epilepsy. Some patients had their first-ever recorded seizure captured on pocEEG, and seven patients without prior epilepsy history were diagnosed with focal epilepsy on subsequent standard EEG (Table 2).

**Table 3:** Overview of grouped diagnoses (*N* = 62)

		Count (%)
Epilepsy and seizure disorders (*n* = 20)	Epilepsy Focal seizure in focal epilepsy Epilepsy, frontal lobe Infantile epilepsy Neonatal epileptic encephalopathy SeLECTS Nonprovoked seizures Neonatal seizures Tonic seizures, Kleefstra syndrome	11 (14.5) 2 (3.2) 1 (1.6) 1 (1.6) 1 (1.6) 1 (1.6) 1 (1.6) 1 (1.6) 1 (1.6)
Status epilepticus (*n* = 19)	Febrile status epilepticus Status epilepticus Nonconvulsive status epilepticus Focal nonconvulsive status epilepticus	8 (12.9) 6 (9.7) 4 (6.5) 1 (1.6)
Febrile and acute seizures (*n* = 11)	Complex febrile seizures Suspected seizures Seizures, gastrointestinal infection Seizures, influenza	4 (6.5) 2 (3.2) 4 (6.5) 1 (1.6)
Neurological disorders (*n* = 8)	Migraine Rasmussen’s encephalitis Vanishing white matter disease Cerebral ischemia CNS infection Hemiparesis Shunt dysfunction	2 (3.2) 1 (1.6) 1 (1.6) 1 (1.6) 1 (1.6) 1 (1.6) 1 (1.6)
Other conditions (*n* = 4)	Breath-holding spells Myoclonia Hypovitaminosis D3 PNES	1 (1.6) 1 (1.6) 1 (1.6) 1 (1.6)

*CNS* central nervous system, *PNES* psychogenic nonepileptic seizures, *SeLECTS* self-limited epilepsy with centrotemporal spikes

### Feasibility and safety of pocEEG implementation

Motion artifacts posed challenges in younger patients and those actively seizing. PED physicians interpreted pocEEG findings cautiously and followed standard care protocols whenever results were inconclusive. When available, the neuropediatrics team provided additional support for pocEEG interpretation and clinical decision-making.

## Discussion

This quality improvement project successfully implemented pocEEG in the PED of a tertiary, academic pediatric hospital and thus enhanced rapid seizure detection, AMS evaluation, and neuromonitoring of critically ill patients. The method integrated well into clinical workflows, with rapid bedside setup, continuous technical improvements, and abnormal findings in 45% of cases. Half of the patients presented outside regular business hours, and in 60% of cases, pocEEG influenced clinical decision making. When results were inconclusive, standard care protocols continued to guide treatment decisions.

### Interpretation

Our results suggest that pocEEG may serve as a valuable adjunct in the PED, particularly for differentiating ongoing seizure activity from non-epileptic events. In some patients with active SE, pocEEG-guided ASM treatment escalation. It also facilitated the exclusion of NCSE in intubated patients and those whose motor seizures had ceased. The observed concordance between pocEEG and follow-up standard EEG supports its potential as a rapid screening tool. The occurrence of some discrepancies emphasizes the need for follow-up standard EEG, particularly in patients with pre-existing conditions. A key strength of this study is its real-world application, which has demonstrated how pocEEG translates into clinical practice by providing rapid diagnostic insights that help decisions to escalate or de-escalate treatment.

### Comparison to existing literature

A recent review summarized the current literature on pocEEG in the PED [14] and highlighted its feasibility [19] and utility in detecting NCSE [11, 12] and its potential to guide more targeted pharmacological treatment [20]. A similar rate of presentations outside business hours has been previously reported [13].

We adopted a previously published method that integrates EEG recording into a standard patient monitor [13]. Other authors have explored other pocEEG setups, including dedicated EEG recording devices and various electrode types and placements [11, 12, 20]. Some used two-channel EEG setups (Fp1/Fp2-P3/P4) [12, 20], and others used four-channel configurations over the frontal (Fp1-A1 and Fp2-A2) and occipital regions (O1-A1 and O2-A2) with additional reference electrodes, resulting in nine electrodes [11]. More channels allow a broader cortical region to be sampled, which increases sensitivity. This has been demonstrated in adult subhairline EEG, where more channels in standard EEG improve detection of abnormalities in intensive care settings [21].

### Impact on practice

These results suggest that pocEEG may support AMS evaluation and detection of NCSE in the PED. Maximizing pocEEG effectiveness requires further standardization of interpretation criteria and improved training for emergency clinicians in EEG assessment. Additionally, optimizing workflows to ensure timely standard EEG follow-up in uncertain cases may improve diagnostic accuracy and patient outcomes.

Although we did not apply the Consolidated Framework For Implementation Research (CFIR) prospectively [22], several CFIR constructs are relevant in retrospect. Strong support from PED and neurophysiology leadership ensured that “intervention characteristics,” “outer setting,” and “inner setting” did not pose major obstacles. However, “knowledge and beliefs” among individuals emerged as a factor critical to successful implementation. For example, after the face-to-face training session, which included seizure pattern videos on patient monitors, PED attending physicians expressed a need for additional basic EEG knowledge. Remote interpretation, as used in other studies [13], was not feasible due to institutional policies, data protection concerns, and reluctance among the neuropediatrics team on call to interpret a pocEEG. To address this gap, we implemented data recording to build a database of normal and pathological pocEEG recordings for educational purposes [23]. Similar approaches have been used successfully in neonatal ICUs, pediatric ICUs [24], and adult EDs [25] to train non-experts in EEG interpretation. This project took an incremental implementation approach that divided the process into manageable steps and thus allowed continuous adaptation and improvement [22].

### Limitations and further directions

Our study has several limitations. First, the relatively small sample size constrains the extent to which our findings can be generalized, and further research with larger cohorts is needed to confirm the clinical utility of pocEEG in pediatric emergency care. Additionally, the study was conducted in a tertiary pediatric center with strong leadership support from both PED and neurophysiology teams, which may not be replicable in other settings. The feasibility of implementing pocEEG in EDs without direct support from the neurophysiology team remains unclear and warrants further investigation. Another limitation is the lack of long-term outcome data and cost-effectiveness analysis. Although pocEEG demonstrated feasibility and clinical impact, its effect on patient outcomes and healthcare resource utilization remains unknown. Future studies should assess whether its use translates into earlier seizure detection, improved treatment outcomes, and reduced hospital stays. The study design also introduces selection bias through its convenience sampling. Recruitment depended on PEM physicians familiar with pocEEG, which may have influenced case selection. Consequently, we cannot use these data to determine the true incidence of SE or NCSE in our PED. Additionally, the liberal use of pocEEG in this QI project was intended to explore its potential applications, but further research should define specific indications and evaluate its limitations in comparison to standard EEG. Lastly, the interpretation of raw EEG tracings by non-experts in the PED remains unexplored. Although our study demonstrated that PED physicians can integrate pocEEG into clinical workflows, the need for further training and standardized interpretation criteria remains a key consideration. Previous studies have emphasized the need for larger, prospective trials to strengthen the evidence base for pocEEG in pediatric emergency care [7, 14, 20].

## Conclusion

This quality improvement study demonstrates that pocEEG is a feasible and valuable tool for rapid neuromonitoring in pediatric emergency care. Its implementation successfully enhanced AMS evaluation and real-time seizure monitoring and integrated effectively into clinical workflows. Although further research is needed to assess its long-term outcomes and cost-effectiveness in larger multicentric cohorts, pocEEG may help bridge diagnostic gaps in settings where 24-h standard EEG availability is limited.

## Supplementary Information

Below is the link to the electronic supplementary material.

Supplementary Material 1 (DOCX 35.2 KB)

## Data Availability

The data that support the findings of this study are available from the corresponding author upon reasonable request.

## Abbreviations

AMS: Altered mental status
ASM: Antiseizure medication
CFIR: Consolidated Framework for Implementation Research
CNS: Central nervous system
ED: Emergency department
EEG: Electroencephalogram
ICU: Intensive care unit
ILAE: International League Against Epilepsy
NCSE: Nonconvulsive status epilepticus
PED: Pediatric emergency department
PEM: Pediatric emergency medicine
PICU: Pediatric intensive care unit
PNES: Psychogenic nonepileptic seizures
QI: Quality improvement
pocEEG: Point-of-care electroencephalogram
SE: Status epilepticus
