# Supplementary material for: Implementation of point-of-care EEG in a pediatric emergency department: a quality improvement study
Source: Eur J Pediatr. 2025 Sep 28;184(10):646. doi: 10.1007/s00431-025-06404-1 (PMC12477078; doi:10.1007/s00431-025-06404-1)
Supplement: Supplementary file 1 — Supplementary Material 1 (DOCX 35.2 KB) [file 431_2025_6404_MOESM1_ESM.docx]

| Supplemental Table 1 – Case details | | | | | | | | | | | | |
| --- | --- | --- | --- | --- | --- | --- | --- | --- | --- | --- | --- | --- |
| Patient # | gender | age (m) | recording duration | epilepsy type | ASM preospital | pocEEG | pocEEG finding | 1st ASM | 2nd ASM | 3rd ASM | sEEG (<48h) | final diagnosis |
| 1 | F | 114 | 20 | Focal Epilepsy | po | Abn | asymmetric background, beta activity, bilat iEDs |  |  |  | R slowing, bilat spikes | Symptomatic epilepsy |
| 2 | M | 167 | 32 | PNES - no epilepsy | iv | N | generalized muscle artifacts, symmetric background, no EDs | iv |  |  | normal | PNES |
| 3 | M | 85 | 8 | Focal Epilepsy | ⎼ | Abn | symmetric background, 4-5 patterns suspicious for iEDs |  |  |  | SeLECTS | SeLECTS |
| 4 | M | 3 | 59 | Focal Epilepsy | ⎼ | Abn | asymmetric background, bilat EDs rhythmic pattern 1,5min seizure | po/rec |  |  | F-T-P-spikes | recurrent seizures |
| 5 | M | 66 | 4 | Focal Epilepsy | rec/po | N | symmetric background, generalized beta activity post ASM |  |  |  | O-slowing bilat | Epilepsy |
| 6 | M | 108 | 30 | Generalized Epilepsy | ⎼ | Abn | symmetric background, generalized iEDs |  |  |  |  | Epilepsy |
| 7 | M | 128 | 14 | No epilepsy | ⎼ | N | mildly asymmetric background, no EDs |  |  |  |  | Migraine, basilar |
| 8 | M | 47 | 10 | Focal Epilepsy | iv | N | symmetric background, artifacts, and generalized beta activity, no EDs | iv |  |  | F-T-P-spikes R | post-SE |
| 9 | M | 0 | 29 | No epilepsy | ⎼ | N | mildly asymmetric background, no EDs |  |  |  |  | Myoclonus |
| 10 | M | 38 | 37 | Focal Epilepsy | rec/po | Abn | asymmetric, rhythmic pattern R; @25min transition into generalized beta activity | iv |  |  |  | NCSE |
| 11 | M | 43 | 12 | acute symptomatic seizure - no epilepsy | ⎼ | N | symmetric background, no EDs |  |  |  | normal | Seizure, GI |
| 12 | M | 160 | 5 | acute symptomatic seizure - no epilepsy | ⎼ | N | mildly asymmetric background, no EDs |  |  |  | normal | 1st seizure |
| 13 | M | 50 | 10 | acute symptomatic seizure - no epilepsy | ⎼ | N | artifacts, symmetric background, no EDs |  |  |  |  | complex FS |
| 14 | M | 120 | 24 | No epilepsy | ⎼ | N | symmetric background, artifacts, no EDs |  |  |  |  | cerebral ischemia |
| 15 | F | 1 | 45 | Focal Epilepsy | ⎼ | Abn | symmetric background, short rhythmic patterns suspicious for iEDs | po/rec |  |  |  | Neonatal epileptic encephalopathy |
| 16 | M | 78 | 25 | Focal Epilepsy | iv | Abn | asymmetric, RDA R, iEDs |  |  |  | SW-R, Sz, | focal SE |
| 17 | M | 1 | 80 | Unknown Epilepsy | ⎼ | Abn | symmetric background, bilateral iEDs |  |  |  | SW-bilat | Neonatal Seizures |
| 18 | F | 97 | 20 | Focal Epilepsy | ⎼ | N | symmetric background, beta activity, no EDs |  |  |  |  | Shunt dysfunction |
| 19 | F | 171 | 118 | acute symptomatic seizure - no epilepsy | ⎼ | N | symmetric background, artifacts, no EDs |  |  |  | C-P-SW-R | Seizure |
| 20 | F | 8 | 31 | No epilepsy | ⎼ | N | symmetric background, artifacts, no EDs |  |  |  |  | Hemiparesis |
| 21 | M | 6 | 41 | Focal Epilepsy | ⎼ | N | symmetric background, no EDs |  |  |  |  | Epilepsy |
| 22 | M | 107 | 140 | Focal Epilepsy | iv | N | symmetric background, intermittent beta activity / artifact, no EDs |  |  |  |  | Epilepsy |
| 23 | M | 50 | 10 | acute symptomatic seizure - no epilepsy | iv | Abn | asymmetric background, beta activity, no EDs |  |  |  | Slowing R | complex FS |
| 24 | M | 8 | 120 | acute symptomatic seizure - no epilepsy | iv | N | symmetric background, no EDs | iv |  |  |  | FSE |
| 25 | F | 12 | 10 | No epilepsy | ⎼ | N | symmetric background, no EDs |  |  |  | normal | breath holding |
| 26 | F | 21 | 110 | acute symptomatic seizure - no epilepsy | ⎼ | N | symmetric background, no EDs | po/rec | po/rec |  | slowing bilat | Seizure, GI |
| 27 | M | 2 | 300 | No epilepsy | ⎼ | N | symmetric background, artifacts, no EDs |  |  |  | C-P-Slowing R | ?Sz |
| 28 | F | 12 | 34 | acute symptomatic seizure - no epilepsy | ⎼ | N | symmetric background, no EDs |  |  |  |  | Seizure, GI |
| 29 | F | 202 | 20 | Focal Epilepsy | iv | Abn | asymmetric background, suspicious for bilateral iEDs | iv |  |  |  | Rasmussen’s encephalitis |
| 30 | F | 207 | 33 | acute symptomatic seizure - no epilepsy | iv | Abn | intermittent asymmetric background, generalized rhythmic pattern (seizure 3 min) | iv |  |  | Beta bilat | SE |
| 31 | M | 50 | 520 | Generalized Epilepsy | ⎼ | Abn | intermittent asymmetric background, ictal-interictal continuum |  |  |  | C-T-P-Sz R | focal NCSE |
| 32 | M | 4 | 95 | Focal Epilepsy | ⎼ | Abn | symmetric background, bilateral EDs |  |  |  | T-P-SW bilat | Infantile Epilepsy |
| 33 | M | 74 | 24 | Focal Epilepsy | ⎼ | Abn | symmetric background, suspicious for iEDs | po/rec |  |  | F-Sz L | Epilepsy, frontal lobe |
| 34 | F | 115 | 87 | Focal Epilepsy | ⎼ | N | symmetric background, artifacts, no EDs |  |  |  |  | Migraine |
| 35 | F | 73 | 34 | acute symptomatic seizure - no epilepsy | ⎼ | Abn | symmetric background, patterns suspicious for iEDs |  |  |  | F-Slowing R, O-SW R | complex FS |
| 36 | F | 7 | 116 | acute symptomatic seizure - no epilepsy | iv | N | intermittent mildly asymmetric, artifacts, no EDs | iv |  |  |  | FSE |
| 37 | F | 22 | 50 | acute symptomatic seizure - no epilepsy | rec/po | Abn | asymmetric, rhythmic discharges R, stop after 40min | po/rec | iv |  |  | FSE |
| 38 | M | 0 | 110 | acute symptomatic seizure - no epilepsy | ⎼ | N | symmetric background, no EDs |  |  |  | normal | HypovitaminD3 |
| 39 | M | 71 | 42 | Focal Epilepsy | ⎼ | N | symmetric background, no EDs |  |  |  | P-O-SW R | Focal seizure |
| 40 | M | 17 | 9 | acute symptomatic seizure - no epilepsy | iv | N | symmetric background, no EDs |  |  |  |  | FSE |
| 41 | F | 8 | 5 | No epilepsy | ⎼ | N | mildly asymmetric background, no EDs |  |  |  | normal | Seizure |
| 42 | F | 37 | 22 | acute symptomatic seizure - no epilepsy | rec/po | N | symmetric background, artifacts, no Eds |  |  |  | normal | Seizure, GI |
| 43 | M | 40 | 75 | Generalized Epilepsy | ⎼ | N | symmetric background, artifacts, no Eds | iv |  |  |  | Epilepsy |
| 44 | M | 11 | 57 | acute symptomatic seizure - no epilepsy | iv | N | intermittent mildly asymmetric, no EDs |  |  |  | intermittent slowing L | CNS Infection |
| 45 | M | 109 | 21 | Focal Epilepsy | ⎼ | N | symmetric background, no EDs |  |  |  |  | Epilepsy |
| 46 | F | 65 | 230 | Generalized Epilepsy | iv | N | symmetric background, artifacts, no EDs | iv | iv |  |  | Epilepsy |
| 47 | M | 41 | 48 | Focal Epilepsy | iv | Abn | intermittent asymmetric background, intermittent R slowing, no EDs |  |  |  | O-Slowing R | FSE |
| 48 | M | 55 | 41 | acute symptomatic seizure - no epilepsy | rec/po | Abn | asymmetric background, artifacts, suspicious for iEDs | po/rec | iv | iv | T-O-Slowing L | SE |
| 49 | M | 19 | 20 | acute symptomatic seizure - no epilepsy | ⎼ | N | symmetric background, artifacts, no EDs | po/rec | po/rec |  | slowing R | FSE |
| 50 | M | 115 | 200 | Generalized Epilepsy | ⎼ | Abn | symmetric background, artifacts, seizure (duration around 1.5min) |  |  |  |  | Epilepsy |
| 51 | M | 76 | 55 | acute symptomatic seizure - no epilepsy | ⎼ | N | symmetric background, no EDs | po/rec |  |  |  | Seizure, Influenza |
| 52 | F | 24 | 50 | Focal Epilepsy | rec/po | Abn | asymmetric background, rhythmic pattern R, intermittent artifacts and rhythmic pattern L | iv |  |  | O-Slowing R | NCSE |
| 53 | M | 118 | 185 | Focal Epilepsy | rec/po | Abn | asymmetric background, focal L and generalized EDs | po/rec | iv | iv |  | Focal seizure |
| 54 | M | 14 | 90 | acute symptomatic seizure - no epilepsy | ⎼ | N | symmetric background, no EDs |  |  |  | normal | complex FS |
| 55 | M | 16 | 61 | acute symptomatic seizure - no epilepsy | iv | Abn | bilat rhythmic EDs, @40min bilat slowing and intermittent generalized beta activity | iv | iv |  | normal | NCSE |
| 56 | F | 70 | 89 | Generalized Epilepsy | rec/po | Abn | symmetric background, generalized and focal EDs, | iv | po/rec |  |  | Epilepsy |
| 57 | F | 9 | 44 | Focal Epilepsy | ⎼ | Abn | symmetric background, artifacts, intermittent generalized beta, possible iEDs L | po/rec |  |  | T-O-Slowing R | Vanishing white matter disease |
| 58 | M | 14 | 60 | acute symptomatic seizure - no epilepsy | ⎼ | N | symmetric background, artifacts, no EDs | po/rec | po/rec | iv | O-slowing L | SE |
| 59 | M | 11 | 62 | Unknown Epilepsy | iv | Abn | bilat rhythmic EDs, @42min bilat slowing and intermittent generalized beta activity | iv | iv |  | slowing, beta | NCSE |
| 60 | F | 10 | 4 | acute symptomatic seizure - no epilepsy | rec/po | Abn | asymmetric background, generalized beta activity, no EDs | iv | po/rec |  | normal | Febrile SE |
| 61 | M | 12 | 15 | Generalized Epilepsy | ⎼ | Abn | asymmetric background, bilat EDs | po/rec | iv |  | SW-bilat | Epilepsy (spasms) |
| 62 | M | 9 | 16 | acute symptomatic seizure - no epilepsy | ⎼ | Abn | intermittent asymmetric background, artifacts, bilateral EDs | po/rec | iv | iv | slowing, delta | SE |
| Abbreviations: ASM – antiseizure medication; Abn – abnormal; Bilat – bilateral; C – central; EDs – epileptic discharges; F – frontal; F-T – fronto-temporal leads (F7/F8-T3/T4); FS – febrile seizure; FSE – febrile status epilepticus; GI – gastrointestinal infection; iEDs – intermittent epileptic discharges ;iv – intravenous; N – normal; NCSE – nonconvulsive status epilepticus; O – occipital; PNES – psychogenic nonepileptic seizures; P – parietal; po/pr – oral/rectal; RDA - rhythmic delta activity; SE – status epilepticus; sEEG – standard EEG; SeLECTS - Self-limited epilepsy with centrotemporal spikes; SW – sharp waves; Sz – seizure / seizure activity; T – temporal | | | | | | | | | | | | |
